# Therapeutic Medication Errors in the Non-Healthcare Facility Setting Associated with Hospital Admission, 2000–2024

**DOI:** 10.1007/s13181-026-01158-0

**Published:** 2026-09-02

**Authors:** Srija Nuguri, Sandhya Kistamgari, Hannah L. Hays, Natalie I. Rine, Nichole L. Michaels, Motao Zhu, Gary A. Smith

**Affiliations:** 1 Center for Injury Research and Policy, The Abigail Wexner Research Institute at Nationwide Children’s Hospital, 700 Children’s Drive, Columbus, OH 43205 USA; 2 Oklahoma State University College of Osteopathic Medicine, Tulsa, OK USA; 3 Department of Pediatrics, The Ohio State University College of Medicine, Columbus, OH USA; 4 Central Ohio Poison Center, Nationwide Children’s Hospital, Columbus, OH USA; 5 Child Injury Prevention Alliance, Columbus, OH USA

**Keywords:** Therapeutic medication error, Hospital admission, Poison, National Poison Data System

## Abstract

**Introduction:**

This study investigated the characteristics and trends of therapeutic medication errors reported to United States (US) poison centers that occurred in a non-healthcare facility setting and were associated with hospital admission.

**Methods:**

Using a retrospective cohort design, National Poison Data System data from 2000 to 2024 were analyzed. US Census Bureau data were used to calculate population-based rates.

**Results:**

There were 115,071 therapeutic medication errors that occurred in a non-healthcare facility setting and were associated with hospital admission reported to US poison centers from 2000 to 2024. Most involved females (55.5%), individuals > 49 years old (56.9%), a single substance (71.5%), or occurred in a residence (96.0%). In addition, 39.0% were admitted to a critical care unit and 53.3% were associated with a serious medical outcome, including moderate effects (45.4%), major effects (7.2%), or death (0.7%, *n* = 745). Cardiovascular drugs (24.7%), analgesics (18.2%), and antidepressants (11.3%) were most commonly involved. The overall hospitalized therapeutic medication error rate per one million US population increased by 196.9% from 7.06 in 2000 to 20.97 in 2024; the rate was highest among adults > 69 years old, females, and errors involving cardiovascular drugs. Of the 115,009 exposures in which at least one therapeutic medication error scenario was reported, the most common scenarios were “other incorrect dose” (23.6%), “wrong medication taken/given” (16.7%), and “inadvertently took/given medication twice” (13.9%).

**Conclusions:**

These study findings highlight opportunities to strengthen prevention efforts targeting therapeutic medication errors, particularly among older adults and individuals using cardiovascular medications, which could meaningfully decrease associated morbidity, mortality, and healthcare costs.

**Supplementary Information:**

The online version contains supplementary material available at https://doi.org/10.1007/s13181-026-01158-0.

## Introduction

Medication errors affect at least 1.5 million people in the United States (US) annually, making them a leading source of preventable injury and fatality [1–5]. Unintentional therapeutic errors associated with medications are defined by America’s Poison Centers as “an unintentional deviation from a proper *therapeutic* regimen that results in the wrong dose, incorrect route of administration, administration to the wrong person, or administration of the wrong substance” [6]. In 2024, there were 361,402 exposures associated with unintentional therapeutic medication errors reported to US poison centers (PCs), including 46 fatalities [7]. Previous research has commonly focused on medication-related therapeutic errors occurring in healthcare facilities [3, 8–11] with fewer studies evaluating those occurring in non-healthcare facility settings [12–19]. Some studies have included exposures both in and outside of healthcare facilities [20–22] or been limited to a specific class of medication [12, 13, 15, 16, 21, 23], age group [12, 18, 19, 22, 23], statewide data [22], non-US data [19], or combined therapeutic medication error exposures with those associated with non-medical use, self-harm, or adverse drug events not involving a therapeutic error [24, 25]. Prior studies of non-healthcare facility-related therapeutic medication errors using the National Poison Data System (NPDS) database have only included data as recent as 2012 [13–16, 18, 20], with the exception of research by DeCoster, et al. [12], which included data through 2021, and most of these investigations evaluated therapeutic errors involving a single class of medication. In addition, the publication by Hodges, et al. [14] is the only prior investigation that focused on serious therapeutic medication errors occurring in the non-healthcare facility setting; that study included exposures associated with moderate effects, major effects, and death from 2000 to 2012. To our knowledge, no previous study has investigated therapeutic medication errors in the non-healthcare setting associated with hospital admission using exposures reported to US PCs involving all classes of medications. Therapeutic medication errors associated with hospital admission are particularly important to study because they consume substantially more healthcare resources and are more likely to be associated with serious clinical outcomes than those managed in outpatient settings or at home.

The objective of this study was to investigate the characteristics and trends of therapeutic medication errors reported to US PCs that occurred in a non-healthcare facility setting and that were associated with hospital admission.

## Methods

### Data Sources

Data reported to the NPDS from January 1, 2000 through December 31, 2024 were analyzed to investigate therapeutic medication errors that occurred in a non-healthcare facility setting and that led to hospital admission. The NPDS is a national database maintained by America’s Poison Centers that includes data from calls to US PCs [26]. Intercensal and postcensal US population estimates from the US Census Bureau were used to calculate population-based rates, including age- and sex-specific rates [27–29]. This study was judged as exempt from approval by the institutional review board at the authors’ institution.

### Case Selection Criteria

This study was limited to unintentional therapeutic errors involving pharmaceutical substances that were directly reported to a PC and did not occur in a healthcare facility. Exposures involving either a single substance or multiple substances that were associated with admission to a critical care unit or non-critical care unit were included in the study as long as the “primary substance” was a pharmaceutical. The primary substance is the substance that was most likely responsible for the observed clinical effects based on the judgement of PC personnel. Exposures that occurred in a healthcare facility, were not admitted, or were admitted to a psychiatric facility were excluded. The NPDS defines healthcare facility as “any hospital-based patient care unit or emergency department, free-standing emergency medical clinic, first aid station, physician’s office, or clinic.” Chronic care/assisted living residential facilities, correctional facilities, detox centers, and nursing homes are not considered healthcare facilities [6]. Exposures involving non-pharmaceutical substances, veterinary substances, diagnostic agents, and radiopharmaceuticals as the primary substance were excluded, as were those with a medical outcome of “confirmed non-exposure” or “unrelated effect.” Additional details about the substance categories included in this study are available from America’s Poison Centers [7]. Deaths reported to the NPDS undergo extensive review and are assigned a “relative contribution to fatality” classification: (1) undoubtedly responsible, (2) probably responsible, (3) contributory, (4) probably not responsible, (5) clearly not responsible, or (6) unknown. America’s Poison Centers considers a fatality to be exposure-related if the substance exposure is judged to be at least contributory (i.e., relative contribution to fatality 1–3) [7]. Fatalities were included in this study if the relative contribution to fatality of the exposure was determined to be “undoubtedly responsible,” “probably responsible,” “contributory,” or “unknown.”

### Study Variables

Variables for this study included sex, age group, exposure site, exposure type (single-substance or multiple-substance), chronicity of the exposure, highest level of health care received, medical outcome, major pharmaceutical category, therapeutic medication error scenario, related clinical effects, and therapies performed. Age was grouped into < 6 years, 6–19 years, 20–49 years, 50–69 years, > 69 years, and unknown. Exposure site included (1) residence, (2) other (includes all other known non-residence, non-healthcare facility exposure sites), and (3) unknown. The NPDS categorizes chronicity of exposure as (1) acute (a single, repeated, or continuous exposure occurring over a period of eight hours or less), (2) acute-on-chronic (a single exposure that was preceded by a continuous, repeated, or intermittent exposure occurring over a period exceeding eight hours), (3) chronic (a continuous, repeated, or intermittent exposure to the same substance lasting longer than eight hours), and (4) unknown [6].

Highest level of health care received is grouped by the NPDS as (1) admitted to a critical care unit, and (2) admitted to a non-critical care unit. The NPDS categorizes medical outcome as (1) no effect, (2) minor effect (minimal symptoms that generally resolve rapidly), (3) moderate effect (more pronounced, prolonged, or systemic symptoms than minor effect), (4) major effect (symptoms that are life-threatening or result in substantial disability or disfigurement), (5) death, (6) not followed (includes judged as nontoxic exposure or minimal clinical effects possible), and (7) unknown (includes unable to follow, judged as a potentially toxic exposure) [6]. Moderate effect, major effect, and death were combined into a single category of “serious medical outcome” during analyses.

For multiple-substance exposures, the major pharmaceutical category was assigned based on the primary substance. Variable categories for therapeutic medication error scenario, related clinical effects, and therapies performed were those used in the NPDS [6].

### Statistical Analysis

IBM SPSS Statistics 29.0 (IBM Corporation, Armonk, NY) and SAS 9.4 (SAS Institute, Inc., Cary, NC) were used for data analyses. SPSS was used for the descriptive statistics presented in Table 1 and Appendices 1 and 2; SAS was used for all other analyses. Cases with an unknown value for a variable were excluded from the denominator when calculating percentages for that variable. Consistent with our retrospective cohort study design, unadjusted risk ratios (RRs) with 95% confidence intervals (CIs) were calculated to assess the magnitude of the relationships between key characteristics (e.g., age group, exposure chronicity, and exposure type) and outcomes (e.g., highest level of health care received and medical outcome).

**Table 1:** Characteristics of therapeutic medication errors associated with hospital admission reported to United States poison centers by age group, 2000–2024

Age group																
Characteristics		< 6 years		6–19 years		20–49 years			50–69 years			> 69 years		Unknown		Total
		n (%)^a^		n (%)^a^		n (%)^a^			n (%)^a^			n (%)^a^		n		n (%)^a^
Sex																
Male		5,697 (57.4)		7,936 (58.5)		10,323 (40.5)			13,996 (43.3)			12,710 (39.0)		455		51,117 (44.5)
Female		4,221 (42.6)		5,628 (41.5)		15,176 (59.5)			18,319 (56.7)			19,843 (61.0)		599		63,786 (55.5)
Unknown		49		11		17			9			11		71		168
Exposure type																
Single-substance		9,010 (90.4)		10,795 (79.5)		19,283 (75.6)			22,836 (70.6)			19,372 (59.5)		930		82,226 (71.5)
Multiple-substance		957 (9.6)		2,780 (20.5)		6,233 (24.4)			9,488 (29.4)			13,192 (40.5)		195		32,845 (28.5)
Exposure site																
Residence		9,712 (97.9)		13,056 (96.7)		24,248 (95.7)			30,861 (95.9)			30,915 (95.2)		1,083		109,875 (96.0)
Other		207 (2.1)		452 (3.3)		1,077 (4.3)			1,306 (4.1)			1,561 (4.8)		31		4,634 (4.0)
Unknown		48		67		191			157			88		11		562
Exposure chronicity																
Acute		6,434 (65.0)		8,157 (60.6)		10,894 (43.4)			13,716 (43.2)			15,081 (47.1)		661		54,943 (48.5)
Acute-on-chronic		2,304 (23.3)		4,122 (30.6)		9,033 (36.0)			13,049 (41.1)			13,121 (41.0)		337		41,966 (37.0)
Chronic		1,159 (11.7)		1,190 (8.8)		5,175 (20.6)			4,968 (15.7)			3,786 (11.8)		102		16,380 (14.5)
Unknown		70		106		414			591			576		25		1,782
Medical outcome																
No effect		2,797 (28.5)		3,032 (22.7)		3,773 (15.1)			5,698 (17.9)			6,385 (19.9)		261		21,946 (19.4)
Minor effect		2,510 (25.6)		3,684 (27.6)		6,873 (27.6)			7,218 (22.7)			6,315 (19.7)		193		26,793 (23.7)
Serious medical outcome^b^		3,773 (38.4)		6,172 (46.2)		13,435 (53.9)			18,046 (56.8)			18,460 (57.5)		314		60,200 (53.3)
Moderate effect		3,158 (32.2)		5,550 (41.6)		11,289 (45.3)			15,271 (48.0)			15,731 (49.0)		282		51,281 (45.4)
Major effect		581 (5.9)		607 (4.5)		2,014 (8.1)			2,565 (8.1)			2,377 (7.4)		30		8,174 (7.2)
Death		34 (0.3)		15 (0.1)		132 (0.5)			210 (0.7)			352 (1.1)		2		745 (0.7)
Not followed^c^		736 (7.5)		457 (3.4)		833 (3.3)			863 (2.7)			950 (3.0)		195		4,034 (3.6)
Unknown^d^		151		230		602			499			454		162		2,098
Highest level of health care received																
Admitted to critical care unit		3,967 (39.8)		4,925 (36.3)		9,611 (37.7)			13,098 (40.5)			12,881 (39.6)		417		44,899 (39.0)
Admitted to non-critical care unit		6,000 (60.2)		8,650 (63.7)		15,905 (62.3)			19,226 (59.5)			19,683 (60.4)		708		70,172 (61.0)
Total (Row %)		**9**,**967 (8.7)**		**13**,**575 (11.9)**		**25**,**516 (22.4)**			**32**,**324 (28.4)**			**32**,**564 (28.6)**		**1**,**125**		**115**,**071 (100.0)**

^a^ Column percentages may not add to 100.0% due to rounding error

^b^ Includes moderate effect, major effect, and death

^c^ Includes not followed (minimal clinical effects possible) and not followed (judged as a non-toxic exposure)

^d^ Includes unable to follow (judged as a potentially toxic exposure)

## Results

There were 115,071 therapeutic medication error-related exposures in the non-healthcare facility setting reported to US PCs from 2000 to 2024 associated with hospital admission (Table 1). Most exposures involved females (55.5%), individuals > 49 years old (56.9%), a single substance (71.5%), or occurred at a residence (96.0%).

### Chronicity of Exposure

Most exposures were acute (48.5%), followed by acute-on-chronic (37.0%) and chronic (14.5%) (Table 1). This pattern was consistent across all age groups. However, the proportion of acute exposures decreased with an increase in age group from 65.0% among children < 6 years old to 47.1% among adults > 69 years old, while the proportion of acute-on-chronic exposures increased with an increase in age group. The proportion of chronic exposures was highest among individuals 20–49 years old (20.6%). The proportions of moderate effects and major effects were higher among chronic exposures (52.9% moderate and 12.6% major) than acute exposures (42.1% moderate and 5.9% major). Compared with acute exposures, acute-on-chronic (unadjusted RR: 1.40, 95% CI: 1.38–1.42) and chronic exposures (unadjusted RR: 1.11, 95% CI: 1.09–1.12) were more likely to be associated with a serious medical outcome.

### Highest Level of Health Care Received

Admissions associated with therapeutic medication errors were to a non-critical care unit in 61.0% of exposures, while 39.0% were admitted to a critical care unit, and these proportions varied little across age groups (Table 1). Multiple-substance exposures were more likely to be associated with admission to a critical care unit (40.9%) (unadjusted RR: 1.05, 95% CI: 1.03–1.06) than single-substance exposures (38.3%) (Appendix 1).

### Medical Outcome

Most (53.3%) exposures involving therapeutic medication errors admitted to a hospital were associated with a serious medical outcome, including those with moderate effects (45.4%), major effects (7.2%), or death (0.7%, *n* = 745) (Table 1). The proportion of serious medical outcomes increased with increase in age group from 38.4% among children < 6 years old to 57.5% among adults > 69 years old. Multiple-substance exposures were more likely to be associated with a serious medical outcome (59.5%) (unadjusted RR: 1.17, 95% CI: 1.16–1.18) than single-substance exposures (50.7%) (Appendix 1). In addition, adults > 19 years old were more likely to experience a serious medical outcome (unadjusted RR: 1.30, 95% CI: 1.29–1.32) than youth 19 years and younger.

Among the 745 fatalities, 57.9% (*n* = 431) were females, 67.1% (*n* = 500) involved a single-substance, and 47.2% (*n* = 352) involved adults > 69 years old. The relative contribution to fatality of the therapeutic medical error-related exposure was determined to be “contributory” for 158 (21.2%) deaths, “probably responsible” for 150 (20.1%), “undoubtedly responsible” for 126 (16.9%), and “unknown” for 311 (41.7%) fatalities. The most common major pharmaceutical category associated with fatality was analgesics (40.9%, *n* = 305), followed by cardiovascular drugs (30.3%, *n* = 226), antineoplastics (3.9%, *n* = 29), hormones and hormone antagonists (3.8%, *n* = 28), and asthma therapies (3.1%, *n* = 23). The most common scenarios associated with fatality were other incorrect dose (30.6%, *n* = 228), health professional/iatrogenic error (pharmacist/nurse) (13.7%, *n* = 102), medication doses given/taken too close together (11.0%, *n* = 82), wrong medication given/taken (6.8%, *n* = 51), and more than one product containing same ingredient (3.9%, *n* = 29).

### Major Pharmaceutical Category

The most common major pharmaceutical category associated with therapeutic medication error-related hospital admission was cardiovascular drugs (24.7%), followed by analgesics (18.2%), antidepressants (11.3%), hormones and hormone antagonists (9.2%), sedative/hypnotics/antipsychotics (8.4%), and anticonvulsants (8.1%) (Appendix 2). Cardiovascular drugs and analgesics were the most common major pharmaceutical categories across all age groups, except 20–49 years, where analgesics and antidepressants were most common. The proportion of serious medical outcomes was highest among exposures involving opioid antagonists (72.0%), followed by muscle relaxants (66.6%), anesthetics (59.6%), cardiovascular drugs (58.0%), and asthma therapies (57.1%). The proportion of admissions to a critical care unit was greatest among anesthetics (63.3%), followed by opioid antagonists (56.6%), muscle relaxants (51.3%), cardiovascular drugs (47.9%), and asthma therapies (46.7%).

### Therapeutic Medication Error Scenario

Of the 115,009 exposures in which at least one therapeutic medication error scenario was reported, the most common scenarios associated with hospital admission were “other incorrect dose” (23.6%), “wrong medication taken/given” (16.7%), “inadvertently took/given medication twice” (13.9%), “inadvertently took/given someone else’s medication” (11.2%), and “health professional/iatrogenic error” (8.2%) (Appendix 3). There was variation in the rank order of these scenarios across age groups.

### Related Clinical Effects and Therapies Performed

Of the 82,346 exposures in which at least one related clinical effect was reported, the most commonly observed effects were drowsiness/lethargy/mild central nervous system depression (31.0%), hypotension (16.9%), bradycardia (15.8%), tachycardia (12.7%), nausea (12.0%), vomiting (11.5%), and confusion (10.7%). Other less common, but clinically important, effects included respiratory depression (4.0%), moderate central nervous system depression (3.2%), major central nervous system depression/coma (2.7%), and seizures (single, multi/discrete, status) (1.9%), asystole/cardiac arrest (0.7%), renal failure (0.7%), respiratory arrest (0.5%), and ventricular tachycardia/ventricular fibrillation/Torsade de pointes (0.3%).

Of the 84,159 exposures in which at least one therapy was performed, the most common therapies were intravenous fluids (58.4%), oxygen (14.4%), single-dose activated charcoal (13.7%), and intravenous or oral acetylcysteine (11.6%). Other less common, but clinically important, therapies included vasopressors (4.9%), mechanical ventilation (3.6%), and non-invasive ventilation (0.4%).

However, there was differential missingness of data for related clinical effects associated with clinical severity. Cases with minor clinical effects were missing documentation of related clinical effects (12.7%) more often than those with moderate effects (5.2%) or major effects (3.4%).

### Secular Trends

**Fig. 1:**
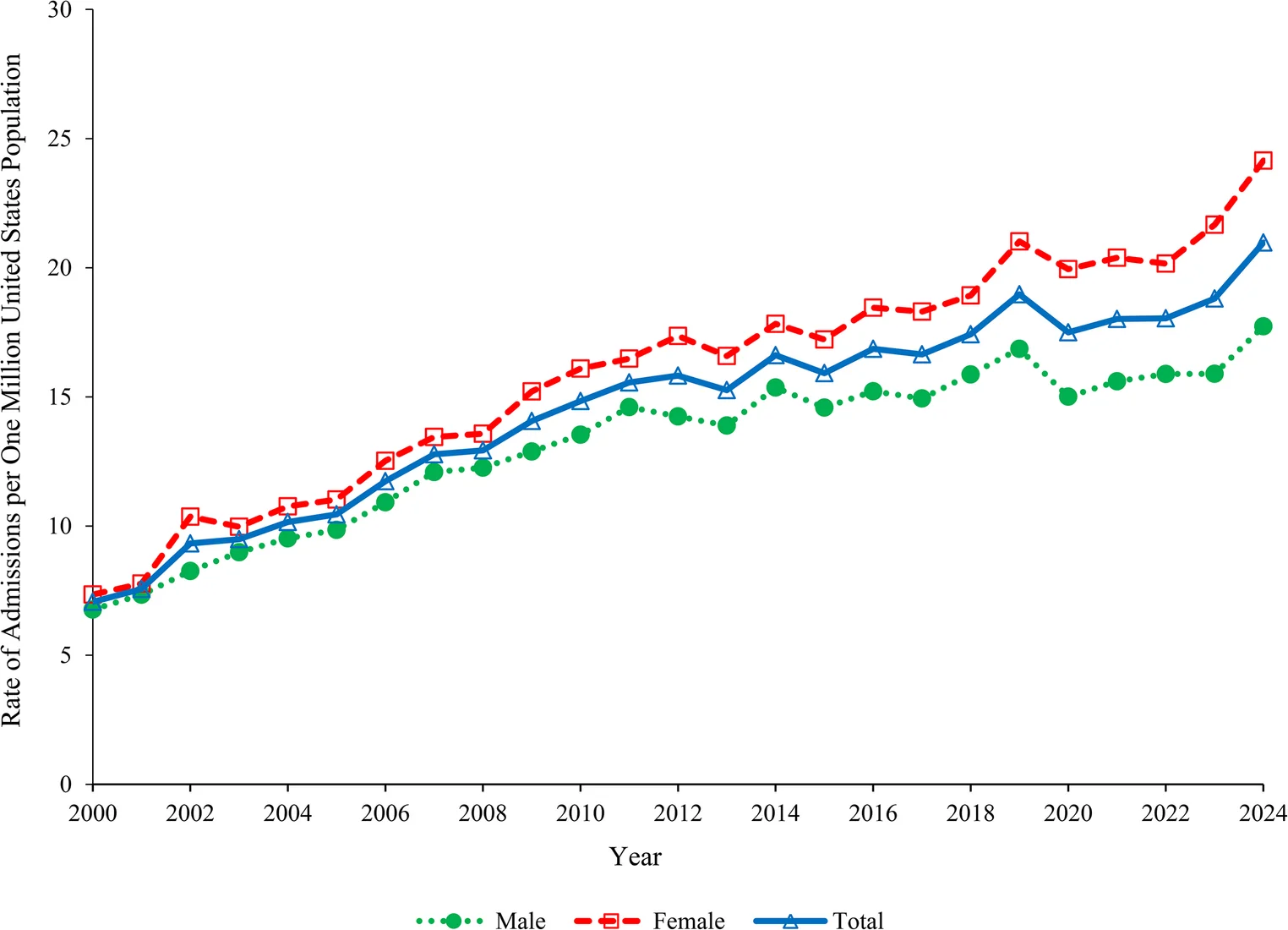
Annual rate per one million United States population of therapeutic medication errors associated with hospital admission reported to United States poison centers by sex, 2000–2024

**Fig. 2:**
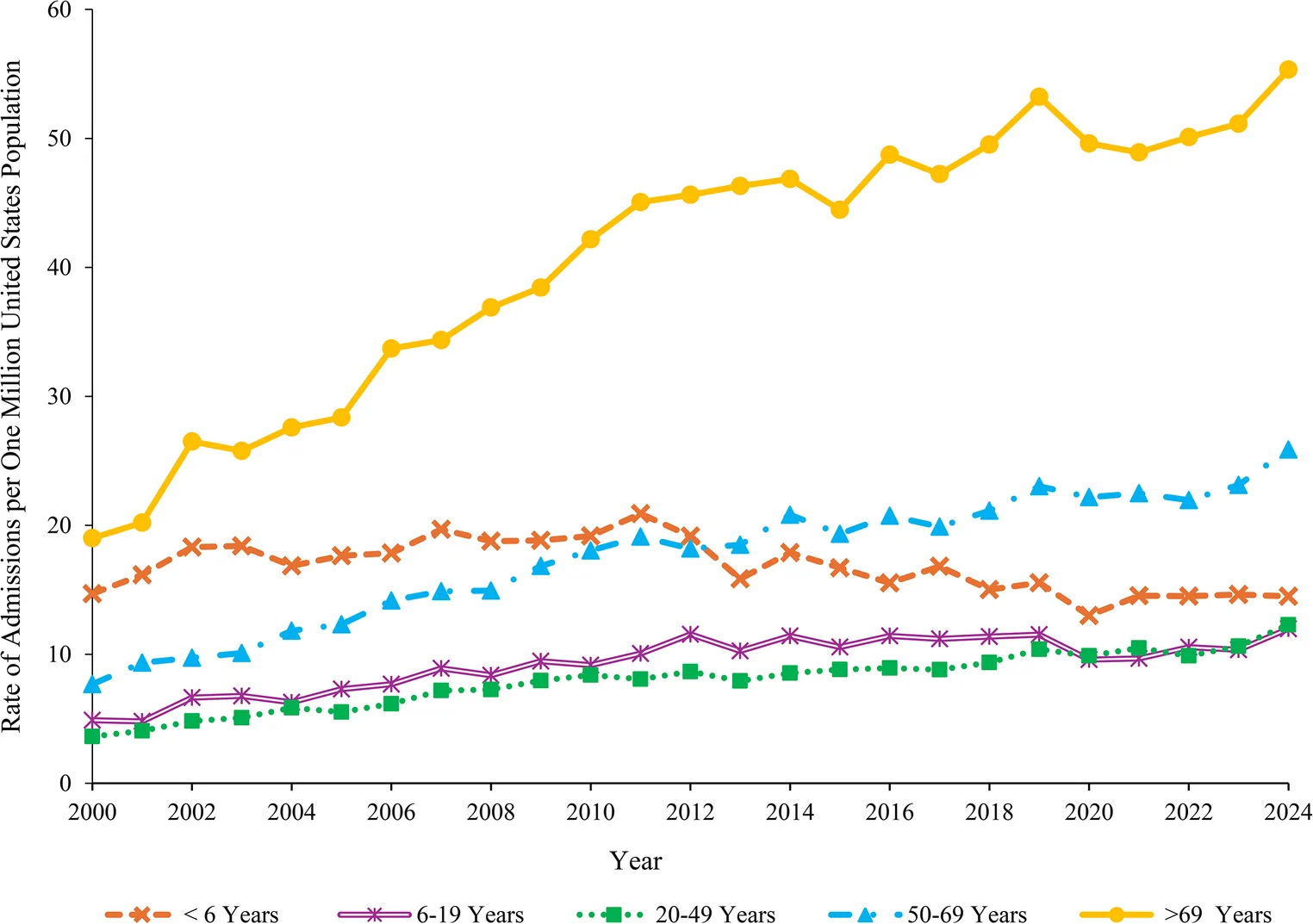
Annual rate per one million United States population of therapeutic medication errors associated with hospital admission reported to United States poison centers by age group, 2000–2024

**Fig. 3:**
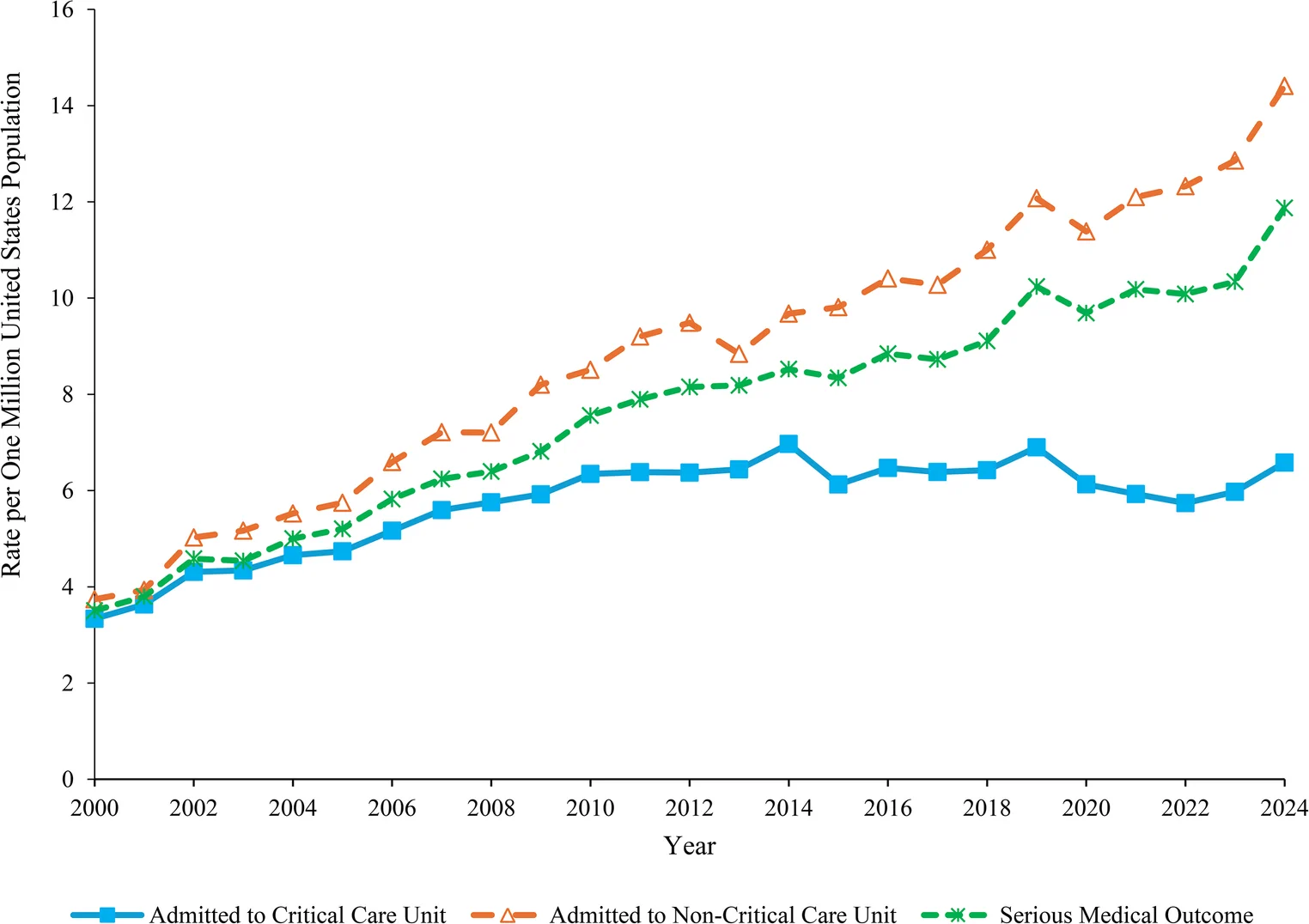
Annual rate per one million United States population of therapeutic medication errors associated with critical care unit admission non-critical care unit admission or a serious medical outcome reported to United States poison centers, 2000–2024

The rate of non-healthcare facility-related therapeutic medication errors associated with hospital admission per one million US population increased by 196.9% from 7.06 in 2000 to 20.97 in 2024 (Fig. 1). The increasing rate trend was seen for all age groups and both sexes except for children < 6 years old, who initially experienced a 42.3% rate increase from 14.69 in 2000 to 20.90 in 2011, followed by a 30.6% rate decrease to 14.50 in 2024 (Fig. 2). The rate was highest among adults > 69 years throughout the study period; it increased by 191.1% from 19.01 in 2000 to 55.34 in 2024. The rate was consistently higher among females than males during the study years (Fig. 1). The rate for females increased by 228.6% from 7.35 in 2000 to 24.15 in 2024 and the rate for males increased by 162.1% from 6.77 in 2000 to 17.73 in 2024. When considering the highest level of health care received, the rate of admissions to a critical care unit increased by 97.3% from 3.34 in 2000 to 6.58 in 2024, while the rate of admissions to a non-critical care unit increased by 285.0% from 3.74 in 2000 to 14.41 in 2024 (Fig. 3). Similarly, the rate of serious medical outcomes increased by 239.0% from 3.50 in 2000 to 11.88 in 2024.

**Fig. 4:**
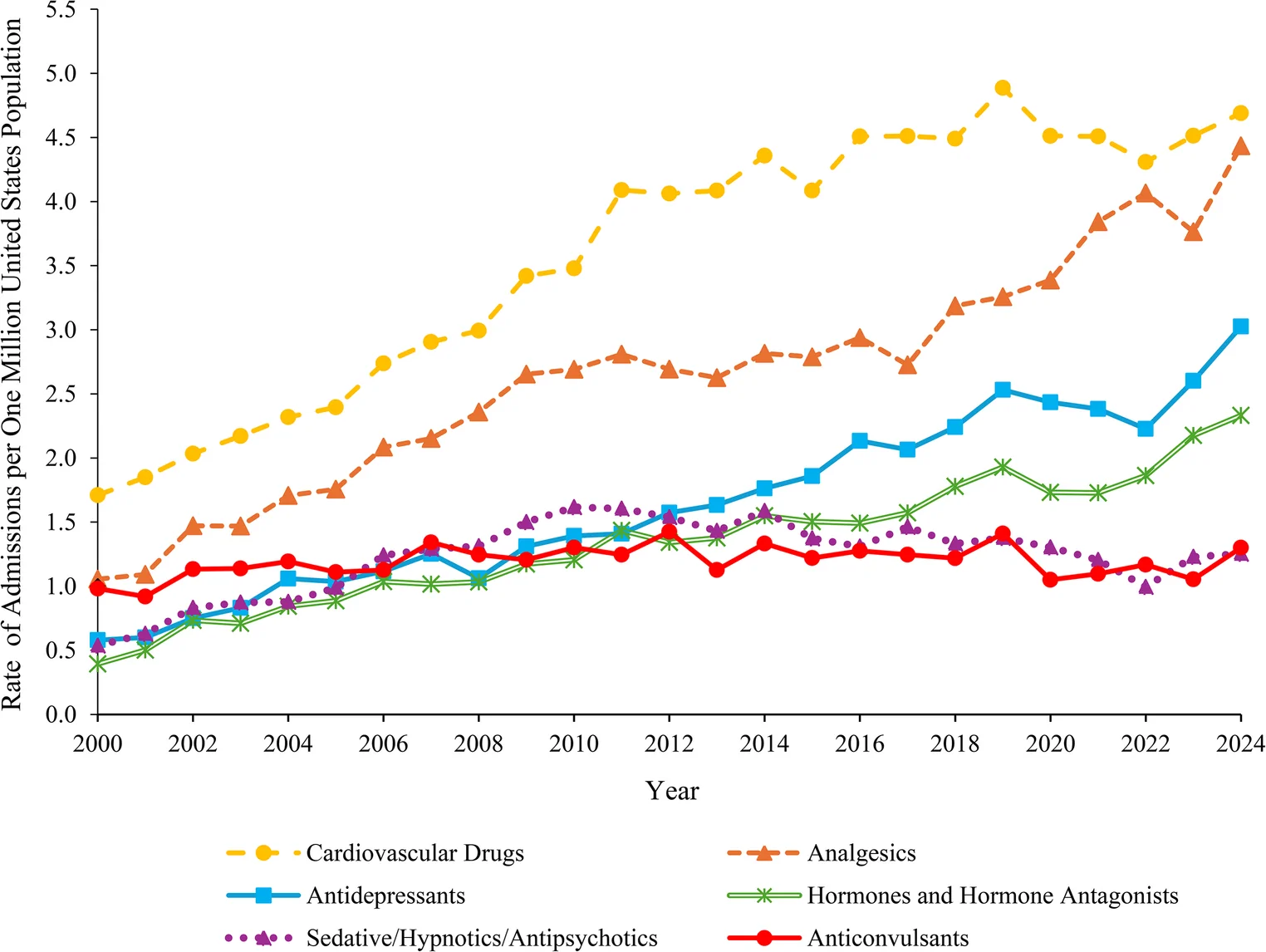
Annual rate per one million United States population of therapeutic medication errors associated with hospital admission reported to United States poison centers by selected major pharmaceutical category, 2000–2024

The rates of therapeutic medication errors associated with hospital admission for the six most common major pharmaceutical categories all increased during the study period. Cardiovascular drugs consistently had the highest rate, increasing by 174.4% from 1.71 in 2000 to 4.69 in 2024 (Fig. 4). Analgesics had the second highest rate across the study years, increasing by 320.1% from 1.06 in 2000 to 4.43 in 2024. Similarly, antidepressants increased by 422.4% from 0.58 in 2000 to 3.03 in 2024, hormones and hormone antagonists increased by 491.1% from 0.39 in 2000 to 2.33 in 2024, and anticonvulsants increased 32.6% from 0.98 in 2000 to 1.30 in 2024 with a maximum of 1.42 in 2012. Sedative/hypnotics/antipsychotics increased by 201.2% from 0.54 in 2000 to 1.62 in 2010 before decreasing by 22.5% to 1.25 in 2024, representing an overall 131.5% increase during the study period. In contrast, the rate for asthma therapies fluctuated over time but decreased overall by 70.2% from 0.22 in 2000 to 0.06 in 2024.

## Discussion

There were more than 115,000 therapeutic medication error-related exposures reported to US PCs from 2000 to 2024 that occurred in the non-healthcare facility setting and were associated with hospital admission, with 96% occurring in a residence. The population-based rate of these admissions increased substantially over time, parallel with ongoing increases in prescription medication use in the US [30]. Rate increases were observed for both critical care (97%) and non-critical care (285%) admissions. Although the increase was greater for non-critical care admissions, nearly 40% of exposures required critical care and more than half were associated with serious medical outcomes, including 0.7% resulting in death, underscoring the clinical severity of many of these exposures. The rate of serious medical outcomes increased continuously over the study period, consistent with the increase previously observed for all (i.e., hospitalized and non-hospitalized) therapeutic medication errors from 2000 to 2012 [14]. These findings reinforce that therapeutic medication errors occurring in the community are not benign events and that the rate of hospital admissions related to these events is increasing.

The rate of hospital admissions related to therapeutic medication errors involving females was higher and increased more rapidly than that for males throughout the study period. In agreement with prior research, this rate trend was primarily influenced by adults 20 years and older, among whom females accounted for approximately 60% of the exposures [14, 20]. The female predominance and widening gap between the sexes suggests an opportunity for targeted prevention efforts. Our study design and the data limitations of the NPDS precluded our ability to determine factors contributing to this trend, which remains an area for future research.

Children < 6 years old experienced a decrease in the rate of therapeutic medication errors associated with hospital admission starting after 2011, which updates the findings of most previous NPDS studies on therapeutic medication errors, which reported data only through 2012 [13–16, 18]. This trend may reflect multifactorial improvements in pediatric medication safety, including regulatory actions and other public health initiatives. Such actions included recommendations against the use of over-the-counter cough and cold products among young children, which was commonly associated with therapeutic medication errors in that age group [18, 23]. Therapeutic medication errors reported to US PCs involving these products decreased among children < 6 years old following recommendations by the US Food and Drug Administration and the Consumer Healthcare Products Association in 2007 and 2008 [31]. In addition, these two groups moved to a single, less concentrated, formulation of pediatric oral liquid acetaminophen in 2011 [32]. Further, the US Food and Drug Administration issued a guidance for industry on dosage delivery devices for orally ingested over-the-counter liquid drug products in 2011 to reduce therapeutic medication errors [33]. These national efforts were supported by the American Academy of Pediatrics, the Prevention of Overdoses and Treatment Errors in Children Taskforce (PROTECT) initiative, and other groups, leading to standardized dosing devices, liquid medication flow restrictors, improved labeling, and caregiver education, which have been shown to reduce therapeutic medication errors [34, 35].

In contrast to young children, adults > 69 years old experienced a 191% increase in the rate of hospital admissions related to therapeutic medication errors that occurred in a non-healthcare facility setting from 2000 to 2024, and they had a higher rate than other age groups across the study years. This finding agrees with prior studies reporting more therapeutic medication errors among older adults [20, 22, 36]. Adults > 69 years old had a notably higher proportion of multiple-substance exposures (40%) than other age groups in this study and a high proportion of acute-on-chronic exposures (41%). Polypharmacy may be a major contributing factor [37]. Complex medication regimens increase the risk of therapeutic medication errors, drug–drug interactions, and confusion regarding administration schedules [38, 39]. Age-related factors such as cognitive impairment, visual limitations, and physical decline further compound this risk [40–42]. Multifaceted prevention interventions are needed, including improvements in medication labeling, simplification of dosing regimens, adherence aids such as pill organizers and reminder systems, and enhanced older adult and caregiver education [43, 44].

Cardiovascular drugs were the most commonly implicated pharmaceutical category, consistent with prior NPDS analyses [14, 15, 20]. They accounted for one-fourth of therapeutic medication errors, and their rate of hospital admission was higher than that for other major pharmaceutical categories, increasing by 174% during the study period. The prominence of cardiovascular medications likely reflects both their widespread use, especially among older adults, and their narrow therapeutic windows, where relatively small deviations in dosing may result in clinically significant toxicity. The increasing rate of cardiovascular drug-related therapeutic errors parallels the growing burden of cardiovascular disease and associated pharmacotherapy in the US [45]. Additionally, cardiovascular disease management often involves complex, multidrug regimens. Limiting the use of multiple medications by introducing single-pill combination therapies may effectively treat cardiovascular disease risks [46], while improving medication adherence and reducing therapeutic medication errors [43, 47].

The three most common therapeutic medication error scenarios overall were “other incorrect dose,” “wrong medication taken/given,” and “inadvertently took/given medication twice,” which aligns with the scenarios found in prior NPDS studies [14, 17, 36]. The rank order of these scenarios varied by age group and “inadvertently took/given someone else’s medication” was also a common scenario, especially among the pediatric age groups. The frequency of pediatric therapeutic medication errors at home has been reported to range from 30 to 80% [48, 49]. These errors frequently involve incorrect measurement of liquid formulations, which is, in part, associated with a lack of understanding by caregivers of weight-based dosing [35, 48] and confusion or miscommunication about dose timing, which is exacerbated by communication barriers encountered with young children [50]. Among adults, therapeutic medication errors more often reflect regimen complexity and timing errors [37–39]. These findings suggest that age-related prevention strategies should be tailored to the predominant types of therapeutic medication error in each group. For young children, interventions should emphasize milliliter-only dosing, use of standardized dosing devices, clear product labeling, and caregiver education. For older adults, interventions should focus on regimen simplification, medication organization and adherence aids (including use of physical or digital logs to record when medications are given), clear product labeling, education for them and their caregivers, and appropriate support for those with cognitive or functional (e.g., vision) impairments.

### Study Limitations

This study has several limitations. It underestimates the number of therapeutic medication errors associated with hospital admission in the US because not all these events are reported to PCs. In addition, because the study was restricted to hospital admissions, serious medical outcomes among non-admitted individuals were not captured. Further, there may be reporting biases that limit generalizability of our findings. Because data are self-reported, PCs and America’s Poison Centers cannot completely verify them. Although cases with an exposure site coded as a healthcare facility were excluded from the study, there remained 562 cases with an unknown exposure site; it is possible that some of these “unknown” cases occurred in a healthcare facility. Misclassification may occur because of self-report, incomplete follow up, or missing, incomplete or inaccurate documentation. In addition, some substances may be more prone to misclassification error regarding the reason for exposure; for example, an adverse reaction being miscoded as a therapeutic error. Determination of the primary substance in multiple-substance exposures is based on the judgement of PC personnel and also may result in misclassification; such misclassification may disproportionately affect older age groups, especially those > 69 years old, among whom multiple-substance exposures were more common. However, misclassification errors are mitigated by extensive training of PC personnel and standardized data protocols and quality checks. Cases with lower clinical severity had a higher proportion of missing data for related clinical effects; therefore, the numbers and proportions of cases reported for the related clinical effects variable should be interpreted with caution. Exposures reported to the NPDS do not necessarily represent a poisoning or overdose. The decision whether to admit a patient to a hospital is affected by other factors besides exposure toxicity, including comorbidities and social factors such as availability and quality of care outside the hospital and concerns about communication or ability to follow up if clinical deterioration were to occur. Therefore, causality between therapeutic medication errors and hospital admission cannot be established because of limitations of the study design and database. Despite its limitations, the NPDS is a longstanding national database that is a valuable resource for the epidemiologic study of substance exposures in the US.

## Conclusions

This study provides important insight into the subset of non-healthcare facility-related therapeutic medication errors that escalate to hospitalization. The substantial and sustained overall increase in hospital admissions associated with therapeutic medication errors occurring in the non-healthcare facility setting over the 24 years of this study suggests that additional prevention efforts are needed. In the context of growing medication use and regimen complexity, clinicians should prioritize appropriate prescribing practices and patient counseling on high-risk medications and common therapeutic medication error scenarios occurring in the non-healthcare facility setting, especially among the groups at higher risk, including older adults, females, and individuals using cardiovascular drugs. Policy makers, government agencies, healthcare organizations, advocates, and manufactures should also expand their efforts to reduce these medication errors. Identifying and addressing factors associated with therapeutic medication errors have the potential to meaningfully decrease preventable morbidity and mortality, as well as healthcare costs, given the considerable healthcare utilization associated with these events.

## Supplementary Information

Below is the link to the electronic supplementary material.

Supplementary Material 1

## Data Availability

Data analyzed in this study were from the National Poison Data System, which is owned and managed by America’s Poison Centers. Data requests should be submitted to America’s Poison Centers.

## Abbreviations

CI: confidence interval
NPDS: National Poison Data System
PC: poison center
RR: risk ratio
US: United States
