# Supplementary material for: Therapeutic Medication Errors in the Non-Healthcare Facility Setting Associated with Hospital Admission, 2000–2024
Source: J Med Toxicol. 2026 Sep 2;22(4):430–40. doi: 10.1007/s13181-026-01158-0 (PMC13633213; doi:10.1007/s13181-026-01158-0)
Supplement: Supplementary file 1 [file 13181_2026_1158_MOESM1_ESM.docx]

| **Appendix 1. Characteristics of Therapeutic Medication Errors Associated with Hospital Admission Reported to United States Poison Centers by Exposure Type, 2000-2024** | | | |
| --- | --- | --- | --- |
|  | **Exposure Type** | |  |
| **Characteristics** | Single-substance | Multiple-substance | Total |
|  | n (%) | n (%) | n (%) |
| **Sex** |  |  |  |
| Male | 36,930 (45.0) | 14,187 (43.2) | 51,117 (44.5) |
| Female | 45,142 (55.0) | 18,644 (56.8) | 63,786 (55.5) |
| Unknown | 154 | 14 | 168 |
|  |  |  |  |
| **Age (years)** |  |  |  |
| <6 | 9,010 (11.1) | 957 (2.9) | 9,967 (8.7) |
| 6-19 | 10,795 (13.3) | 2,780 (8.5) | 13,575 (11.9) |
| 20-49 | 19,283 (23.7) | 6,233 (19.1) | 25,516 (22.4) |
| 50-69 | 22,836 (28.1) | 9,488 (29.1) | 32,324 (28.4) |
| >69 | 19,372 (23.8) | 13,192 (40.4) | 32,564 (28.6) |
| Unknown | 930 | 195 | 1,125 |
|  |  |  |  |
| **Exposure Site** |  |  |  |
| Residence | 79,145 (96.7) | 30,730 (94.0) | 109,875 (96.0) |
| Other | 2,678 (3.3) | 1,956 (6.0) | 4,634 (4.0) |
| Unknown | 403 | 159 | 562 |
|  |  |  |  |
| **Exposure Chronicity** |  |  |  |
| Acute | 38,674 (47.7) | 16,269 (50.4) | 54,943 (48.5) |
| Acute-on-chronic | 29,142 (36.0) | 12,824 (39.7) | 41,996 (37.0) |
| Chronic | 13,181 (16.3) | 3,199 (9.9) | 16,380 (14.5) |
| Unknown | 1,229 | 553 | 1,782 |
|  |  |  |  |
| **Medical Outcome** |  |  |  |
| No effect | 16,651 (20.7) | 5,295 (16.4) | 21,946 (19.4) |
| Minor effect | 19,658 (24.4) | 7,135 (22.0) | 26,793 (23.7) |
| Serious medical outcome^a^ | 40,936 (50.7) | 19,264 (59.5) | 60,200 (53.3) |
| Moderate effect | 34,851 (43.2) | 16,430 (50.7) | 51,281 (45.4) |
| Major effect | 5,585 (6.9) | 2,589 (8.0) | 8,174 (7.2) |
| Death | 500 (0.6) | 245 (0.8) | 745 (0.7) |
| Not followed^b^ | 3,346 (4.2) | 688 (2.1) | 4,034 (3.6) |
| Unknown^c^ | 1,635 | 463 | 2,098 |
|  |  |  |  |
| **Highest Level of Health Care Received** |  |  |  |
| Admitted to critical care unit | 31,461 (38.3) | 13,438 (40.9) | 44,899 (39.0) |
| Admitted to non-critical care unit | 50,765 (61.7) | 19,407 (59.1) | 70,172 (61.0) |
|  |  |  |  |
| **Total (Row %)** | **82,226 (71.5)** | **32,845 (28.5)** | **115,071 (100.0)** |
| ^a^ Includes moderate effect, major effect, and death ^b^ Includes not followed (minimal clinical effects possible) and not followed (judged as a non-toxic exposure) ^c^ Includes unable to follow (judged as a potentially toxic exposure) | | | |
| **Appendix 2. Most Common Major Pharmaceutical Categories Involved in Therapeutic Medication Errors Associated with Hospital Admission Reported to United States Poison Centers by Age Group, 2000-2024** **Age Group** \| \| \| \| \| \| \| \|; \| <6 years \| 6-19 years \| 20-49 years \| 50-69 years \| >69 years \| Unknown \| Total \|; **Major Pharmaceutical Category** \| n (%)^a^ \| n (%)^a^ \| n (%)^a^ \| n (%)^a^ \| n (%)^a^ \| n \| n (%)^a^ \|; Cardiovascular Drugs \| 2,080 (20.9) \| 3,707 (27.3) \| 2,436 (9.6) \| 7,267 (22.5) \| 12,756 (39.2) \| 166 \| 28,412 (24.7) \|; Analgesics \| 1,429 (14.3) \| 1,967 (14.5) \| 7,237 (28.4) \| 6,072 (18.8) \| 4,071 (12.5) \| 150 \| 20,926 (18.2) \|; Antidepressants \| 262 (2.6) \| 1,763 (13.0) \| 4,866 (19.1) \| 4,173 (12.9) \| 1,838 (5.6) \| 146 \| 13,048 (11.3) \|; Hormones and Hormone Antagonists \| 223 (2.2) \| 819 (6.0) \| 1,670 (6.5) \| 3,880 (12.0) \| 3,883 (11.9) \| 147 \| 10,622 (9.2) \|; Sedative/Hypnotics/Antipsychotics \| 821 (8.2) \| 1,503 (11.1) \| 2,334 (9.2) \| 2,740 (8.5) \| 2,168 (6.7) \| 91 \| 9,657 (8.4) \|; Anticonvulsants \| 811 (8.1) \| 1,097 (8.1) \| 3,052 (12.0) \| 2,817 (8.7) \| 1,468 (4.5) \| 100 \| 9,345 (8.1) \|; Muscle Relaxants \| 308 (3.1) \| 270 (2.0) \| 658 (2.6) \| 1,020 (3.2) \| 712 (2.2) \| 26 \| 2,994 (2.6) \|; Antimicrobials \| 538 (5.4) \| 196 (1.4) \| 343 (1.3) \| 497 (1.5) \| 582 (1.8) \| 55 \| 2,211 (1.9) \|; Antihistamines \| 499 (5.0) \| 285 (2.1) \| 412 (1.6) \| 385 (1.2) \| 582 (1.8) \| 20 \| 2,183 (1.9) \|; Other \| 2,996 (30.1) \| 1,968 (14.4) \| 2,508 (9.9) \| 3,473 (10.7) \| 4,504 (13.6) \| 224 \| 15,673 (13.6) \|; **Total (Row %)** \| **9,967 (8.7)** \| **13,575 (11.9)** \| **25,516 (22.4)** \| **32,324 (28.4)** \| **32,564 (28.6)** \| **1,125** \| **115,071 (100.0)** \| ^a^ Column percentages may not add to 100.0% due to rounding error | | | |
|  | | | |
|  |  |  |  |

**Appendix 3. Most Common Therapeutic Medication Error Scenarios Associated with Hospital Admission Reported to United States Poison Centers by Age Group, 2000-2024**

|  |  |  | **Age Group** |  |  |  |  | |
| --- | --- | --- | --- | --- | --- | --- | --- | --- |
|  | <6 years | 6-19 years | 20-49 years | 50-69 years | >69 years | Unknown | Total | |
| **Scenario**^b^ | n (%)^a^ | n (%)^a^ | n (%)^a^ | n (%)^a^ | n (%)^a^ | n | n (%)^a^ | |
| Other incorrect dose | 2,152 (21.6) | 2,560 (18.9) | 6,629 (26.0) | 7,676 (23.8) | 7,938 (24.4) | 235 | 27,190 (23.6) | |
| Wrong medication taken/given | 1,063 (10.7) | 2,496 (18.4) | 4,259 (16.7) | 6,386 (19.8) | 4,817 (14.8) | 177 | 19,198 (16.7) | |
| Inadvertently took/given medication twice | 648 (6.5) | 2,268 (16.7) | 3,192 (12.5) | 4,587 (14.2) | 5,073 (15.6) | 168 | 15,936 (13.9) | |
| Inadvertently took/given someone else’s  medication | 1,324 (13.3) | 2,678 (19.7) | 1,632 (6.4) | 2,850 (8.8) | 4,324 (13.3) | 71 | 12,879 (11.2) | |
| Health professional/iatrogenic error  (pharmacist/nurse) | 1,569 (15.8) | 1,014 (7.5) | 1,858 (7.3) | 2,363 (7.3) | 2,443 (7.5) | 164 | 9,411 (8.2) | |
| Medication doses given/taken too close  together | 217 (2.2) | 851 (6.3) | 2,896 (11.4) | 2,530 (7.8) | 2,402 (7.4) | 63 | 8,959 (7.8) | |
| Patient confused or mentally incompetent | 4 (0.0) | 87 (0.6) | 289 (1.1) | 787 (2.4) | 2,470 (7.6) | 12 | 3,649 (3.2) | |
| 10-fold dosing error | 1,218 (12.2) | 296 (2.2) | 316 (1.2) | 405 (1.3) | 423 (1.3) | 16 | 2,674 (2.3) | |
| Incorrect formulation or concentration given | 520 (5.2) | 409 (3.0) | 489 (1.9) | 571 (1.8) | 454 (1.4) | 31 | 2,474 (2.2) | |
| Confused units of measure | 684 (6.9) | 251 (1.8) | 272 (1.1) | 365 (1.1) | 480 (1.5) | 14 | 2,066 (1.8) | |
| More than one product containing same  ingredient | 52 (0.5) | 178 (1.3) | 802 (3.1) | 513 (1.6) | 277 (0.9) | 13 | 1,835 (1.6) | |
| Other | 1,346 (13.6) | 1,711 (12.5) | 5,278 (20.7) | 6,627 (20.5) | 6,391 (19.7) | 289 | 21,642 (18.7) | |
| **Total with at least one scenario recorded** | **9,955** | **13,568** | **25,500** | **32,310** | **32,551** | **1,125** | **115,009** | |
| ^a^ Column percentages used the number of individuals in that column who had at least one recorded scenario as the denominator. The denominator for each column appears as the last entry in the column. Column percentages sum to >100.0% because some individuals had more than one scenario recorded.  ^b^ Scenario category labels are those used by the National Poison Data System | | | | | | | |  |
|  | | | | | | | |  |
